# Evidence for involvement of the alcohol consumption *WDPCP* gene in lipid metabolism, and liver cirrhosis

**DOI:** 10.1038/s41598-023-47371-7

**Published:** 2023-11-23

**Authors:** Felix O’Farrell, Benjamin Aleyakpo, Rima Mustafa, Xiyun Jiang, Rui Climaco Pinto, Paul Elliott, Ioanna Tzoulaki, Abbas Dehghan, Samantha H. Y. Loh, Jeff W. Barclay, L. Miguel Martins, Raha Pazoki

**Affiliations:** 1 Cardiovascular and Metabolic Research Group, Division of Biosciences, Department of Life Sciences, College of Health and Life Sciences, Brunel University, London, UB8 3PH UK; 2 The Francis Crick Institute, London, NW1 1AT UK; 3 Department of Epidemiology and Biostatistics, School of Public Health, St Mary’s Campus, Imperial College London, Norfolk Place, London, W2 1PG UK; 4 UK Dementia Research Institute, Imperial College London, Exhibition Road, London, SW7 2AZ UK; 5 MRC Centre for Environment and Health, Department of Epidemiology and Biostatistics, School of Public Health, St Mary’s Campus, Imperial College London, Norfolk Place, London, W2 1PG UK; 6 British Heart Foundation Centre of Research Excellence, Imperial College London, Du Cane Road, W12 0NN UK; 7 National Institute for Health Research, Imperial Biomedical Research Centre, Imperial College London, Exhibition Road, London, SW7 2AZ UK; 8 Health Data Research UK at Imperial College London, Exhibition Road, London, SW7 2AZ UK; 9 Centre for Systems Biology, Biomedical Research Foundation, Academy of Athens, Athens, Greece; 10 MRC Toxicology Unit, University of Cambridge, Gleeson Building, Tennis Court Road, Cambridge, CB2 1QR UK; 11 Department of Molecular Physiology and Cell Signalling, Institute of Systems, Molecular and Integrative Biology, University of Liverpool, Liverpool, L69 3BX UK; 12 Division of Biomedical Sciences, Department of Life Sciences, College of Health and Life Sciences, Brunel University, London, UB8 3PH UK

**Keywords:** Genetics, Biomarkers, Diseases, Health care, Molecular medicine

## Abstract

Biological pathways between alcohol consumption and alcohol liver disease (ALD) are not fully understood. We selected genes with known effect on (1) alcohol consumption, (2) liver function, and (3) gene expression. Expression of the orthologs of these genes in Caenorhabditis elegans and Drosophila melanogaster was suppressed using mutations and/or RNA interference (RNAi). In humans, association analysis, pathway analysis, and Mendelian randomization analysis were performed to identify metabolic changes due to alcohol consumption. In *C. elegans*, we found a reduction in locomotion rate after exposure to ethanol for RNAi knockdown of ACTR1B and MAPT. In *Drosophila*, we observed (1) a change in sedative effect of ethanol for RNAi knockdown of WDPCP, TENM2, GPN1, ARPC1B, and SCN8A, (2) a reduction in ethanol consumption for RNAi knockdown of TENM2, (3) a reduction in triradylglycerols (TAG) levels for RNAi knockdown of WDPCP, TENM2, and GPN1. In human, we observed (1) a link between alcohol consumption and several metabolites including TAG, (2) an enrichment of the candidate (alcohol-associated) metabolites within the linoleic acid (LNA) and alpha-linolenic acid (ALA) metabolism pathways, (3) a causal link between gene expression of WDPCP to liver fibrosis and liver cirrhosis. Our results imply that WDPCP might be involved in ALD.

## Introduction

Alcohol consumption is a major public health concern and is responsible for over 5% of the global burden of disease^1^. It has been known for a long time that excessive drinking leads to a range of molecular changes including acceleration of hepatic lipogenesis which leads to liver pathologies, such as liver fibrosis and liver cirrhosis commonly known as alcoholic liver disease (ALD). Although some of the known alcohol behavior genes such as *ADH* and *CYP2E1* genes have a known role in hepatic lipogenesis^2^, the full picture of the biological pathways and molecular changes occurring as a result of alcohol consumption that leads to hepatic lipogenesis is not fully understood.

Advances in omics such as genomics and metabolomics within the last two decades have resulted in a boost in our understanding of the mechanism of diseases through agnostic approaches such as genome-wide association studies (GWAS) that revealed numerous genetic loci linked to complex diseases. Recently, we have applied GWAS to identify genetic variants in the form of single nucleotide polymorphisms (SNPs) that are associated with alcohol consumption^3^ as well as with circulating liver enzymes^4^ in the European populations. Some of the identified alcohol genes (e.g., *ADH*, *KLB*, *DRD2*) have been investigated for a better understanding of their involvement in alcohol consumption and health consequences such as hepatic lipogenesis. However, the biological effect of most of the identified alcohol-associated genes remains to be elucidated.

In this study, we aimed to shed light on the biological pathways and molecular changes linking alcohol consumption and liver pathologies. We first investigated molecular consequences of alcohol consumption and the pathways involved at metabolic level using omics approaches. Subsequently in search for common pathways between alcohol consumption and liver pathologies, we identified candidate genes with effect on both alcohol consumption and liver function and then investigated the biological effect of candidate genes in model organism i.e., ethanol-exposed *C. elegans* and *Drosophila* to generate knowledge that could ultimately be used to better understand alcohol related behavior and hepatic lipogenesis. We finally returned to use data from humans to further validate the most plausible candidate genes with evidence of potential involvement in lipogenesis.

## Methods

### Population

**Figure 1:**
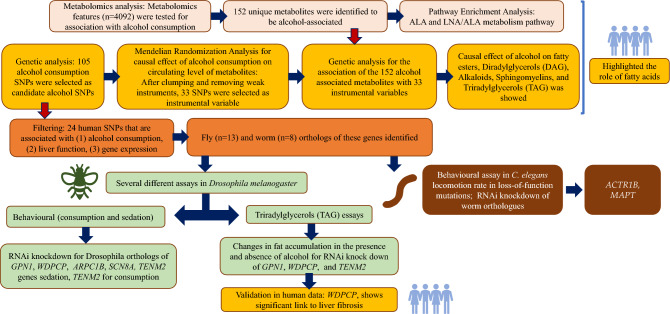
Overview of the study design and the findings.

In the current study, we followed a multi-stage approach using population-based studies and model organisms to better understand pathways involved in alcohol consumption and its health consequences (Fig. 1). We used data from the Airwave Health Monitoring Study^5^, an occupational cohort of 53,116 police officers and staff ages 18 years and over across the UK (Supplementary Table 1). The Airwave Health Monitoring Study was approved by the UK National Research Ethics Service (NRES) North West—Haydock Regional Ethics Committee (REC reference: NRES/19/NW/0054; IRAS ID: 259978). The North-West Haydock REC approved the study protocol and all study documentation, and prior individual informed consent was obtained from each of the study participants. All methods were carried out in accordance with relevant guidelines and regulations. Participants were informed about the study and provided informed consent. Detailed information about the Airwave population, metabolic assays, data processing, metabolite annotation as well as genotyping and imputation is included in the “Supplementary Methods”.

#### Alcohol consumption

Alcohol consumption during the last seven days was assessed through a self-reported questionnaire in the Airwave study. The participants were asked to quantify (1) the number of glasses (small/125 ml) of red wine, white wine/champagne, fortified wine (includes sherry, port and vermouth), (2) the number of pub measures of spirits/liqueurs (includes whisky, gin, rum, vodka and brandy), and (3) the number of pints of beer or cider (include bitter, lager, stout, ale and Guinness) consumed in the last seven days. We considered the density of alcohol to be 0.79 g/ml at room temperature and calculated the total amount of alcohol consumed by summing up the grams of alcohol consumed from each category of alcoholic drink.

#### Analysis of the metabolome

We analyzed the association between alcohol consumption and circulating metabolites within the Airwave sample to identify candidate metabolites associated with alcohol consumption within the Airwave sample (alcohol-associated metabolites). Metabolomics features (n = 4092) were obtained from data acquired by the National Phenome Centre (NPC) using liquid-chromatography/mass spectrometry), covering a wide range of hydrophilic and lipid metabolite classes. We excluded unannotated features leaving 986 annotated metabolites for the analysis and tested the association of these metabolites with alcohol consumption. Subsequently, to assess if the resulting alcohol-associated metabolites were additionally involved in known metabolic pathways, we performed pathway enrichment analysis using all annotated alcohol-associated metabolomics features. To this end, Kyoto Encyclopedia of Genes and Genomes (KEGG)^6^ and The Small Molecule Pathway Database (SMPDB)^7^ were used and the statistical significance level was claimed using a *P*-value calculated using false discovery rate (Pfdr).

#### Genotyping and imputation

In the Airwave study^5^, genotyping was conducted using Illumina Infinium HumanExome-12v1-1 BeadChip Array. QC steps were performed to remove any samples with high missingness (> 3%) or outlier heterozygosity rates (> 3SD from the batch mean), duplicates (ID-based or genotype-based) or presented with high degree of relatedness. Markers with genotype call rate < 98%, significant deviation from Hardy–Weinberg equilibrium (P < 1 × 10^–5^), and low MAF (< 1%) were removed. Genotype data was imputed with 1000 Genomes phase 3 reference panel.

#### GWAS on metabolomics

We performed genome-wide association studies (GWAS) in the Airwave Study to obtain genetic association estimates for alcohol-related SNPs and selected metabolomic features. Untargeted Mass-Spectrometry (MS) was performed in heparin plasma samples of the Airwave Study, producing three separate datasets, namely hydrophilic interaction liquid chromatography (HILIC) positive (HPOS), lipid positive (LPOS), and lipid negative (LNEG). For each metabolomic dataset, we used principal component analysis (PCA) to identify outlier samples and exclude them. The data was residualized using the first ten principal components to account for population stratification. We transformed the data into z-scores, performed individually for each metabolomic feature, using median and median absolute deviations (MAD). Values that were more than 5 MAD from the median were removed and imputed using k-nearest neighbors’ imputation^8^. GWAS were conducted for each metabolomic feature with adjustment for age and sex (N = 1970). Due to the high number of metabolomic features, GWAS were performed using the high-dimensional association analyses (HASE) framework, which applies matrix operation and removes redundant calculation in high-dimensional association analyses to improve computational efficiency^9^.

We used the results from association analyses between the alcohol-associated metabolites and the known alcohol-associated SNPs to further perform a causal inference analysis on these results using the inverse variance weighted two-sample Mendelian randomization (MR) analysis^10^. The aim of the MR analysis was to identify changes in circulating metabolites caused by alcohol consumption. MR is a causal assessment method used in observational studies to mimic randomized controlled trials (RCTs) by taking advantage of the random assortment of alleles at conception. MR uses instrumental variables (i.e., genotype status), that are robustly associated with an exposure of interest, as a natural randomization tool occurring at conception^11^. The detailed methods for selection of alcohol consumption instruments have been described previously^10,12^. Briefly, we obtained genetic association statistics (*β* values) for 105 alcohol-associated SNPs^3,13^ from Liu and colleagues^13^. Clumping and removing weak instruments as described previously^10,12^ ensured the most robust instruments were used for the MR analysis (n = 33).

#### Gene selection for model organisms

We selected a list of 105 alcohol-related SNPs from recently conducted GWAS of alcohol consumption^3,13^. SNPs were selected if they presented a *P*-value lower than a GWAS significance threshold of 5 × 10^−8^ in their association with alcohol consumption. As we were interested in finding pathways involved in hepatic lipogenesis as a result of alcohol consumption, we sought candidate SNPs that showed strong effect with both alcohol consumption and liver function, using our recently published GWAS of circulating liver enzymes^4^. To account for multiple testing, a corrected *P*-value threshold of < 0.00048 was used for the association with liver enzymes. This *P*-value threshold corresponds to a nominal *P*-value (0.05) that has been adjusted for the number of alcohol-related SNPs (n = 105) using the Bonferroni method^14^. SNPs (n = 43; Supplementary Table 2) that were associated with at least one of the three liver enzymes alanine transaminase (ALT), alkaline phosphatase (ALP), and gamma-glutamyl transferase (GGT) were shortlisted to assess their link to gene expression using the GTEx database^15^. We eventually selected SNPs (n = 24) that demonstrated evidence of a statistically significant effect on gene expression of their nearest genes (a cis-eQTL effect; Table 1).

**Table 1:** Overview of the genetic variants with effect on alcohol consumption, liver enzymes and gene expression.

Alcohol SNP *	Annotated gene	eQTL tissue	eQTL effect size§ (effect allele)	eQTL P value^†^	*Drosophila* ortholog^‡^	FlyBase score out of 15§	*C.* *elegans* ortholog (score)^‡^	*Drosophila* strains	*C. elegans* strain
rs823114	*NUCKS1*	Thyroid	− 0.16 (A)	5.80 × 10^–18^	*–*	–	*–*	–	*–*
rs1260326	*GCKR*	Thyroid	0.22 (C)	1.80 × 10^–08^	*–*	–	*–*	–	*–*
rs2178197	*GPN1*	Brain-Cerebellar Hemisphere	− 0.31 (G)	6.50 × 10^–08^	*cg3704*	13	*gop-2 (9) *^*¶*^	*CG3704* (55,294)	RG5036 *gop-2 (gk5528)*,
rs13032049	*WDPCP*	Adipose-Subcutaneous	− 0.16 (G)	3.00 × 10^–04^	*frtz*	13	*–*	*Frz* (55,649)	*–*
rs11692435	*ACTR1B*	Thyroid	0.89 (A)	1.50 × 10^–80^	*arp1*	12	*arp-1 (8) *^*¶*^	Lethal, not studied	FX4735 *arp-1 (tm4735)*
rs12646808	*MSANTD1*	Thyroid	− 0.25 (T)	1.30 × 10^–09^	*cg18766*	8	*–*	–	*–*
rs11940694	*KLB*	Liver	0.22 (A)	4.00 × 10^–05^	*cg9701*	6	*klo-1 (5), klo-2 (5)*	–	Not studied
rs1229984	*ADH1B*	Esophagus-Gastroesophageal Junction	− 1.4 (C)	1.90 × 10^–16^	*fdh*	7	*adh-5 (5)*	–	Not studied
rs10078588	*TENM2*	Thyroid	0.29 (A)	2.80 × 10^–15^	*ten-m*	13	*ten-1 (10) *^*¶*^	*TenM* (29,390)	VC518 *ten-1 (ok641)*,
rs34060476	*MLXIPL*	Pancreas	0.51 (G)	4.50 × 10^–18^	*mondo*	12	*mml-1 (9)*	*Mondo* (27,059)	RB954 *mml-1 (ok849*)
rs10249167	*ARPC1B*	Thyroid	0.3 (G)	1.80 × 10^–25^	*arpc1*	13	*arx-3 (10) *^*¶*^	*Arpc1* (31,246)	VC3166 *arx-3 (ok1122)*
rs988748	*BDNF*	Brain–Nucleus accumbens basal ganglia	0.22 (G)	9.70 × 10^–06^	*–*	–	*–*	–	*–*
rs2071305	*MYBPC3*	Whole blood	− 0.13 (C)	2.30 × 10^–11^	*hbs*	1	*–*	–	*–*
rs7121986	*DRD2*	Esophagus–Muscularis	0.26 (C)	3.50 × 10^–08^	*dop2r*	9	*dop-2 (6), dop-3 (6)*	–	Not studied
rs10876188	*SLC4A8*	Cells-Cultured fibroblasts	− 0.12 (T)	1.80 × 10^–07^	*ndae1*	12	*abts-1 (9)*	Lethal, not studied	RB1381 *abts-1 (ok1566)*
rs7958704	*SCN8A*	Nerve–Tibial	− 0.25 (C)	2.90 × 10^–10^	*para*	12	*cca-1 (1), unc-77 (1), egl-19 (4)*	*Para* (33,923)	JD21 *cca-1 (ad1650)*
rs12312693	*STAT6*	Brain–Frontal Cortex BA9	0.23 (C)	8.00 × 10^–05^	*stat92e*	9	*sta-2 (4), sta-1 (7)*	–	RB796 *sta-1 (ok587)*
rs11625650	*KIF26A*	Whole blood	− 0.26 (A)	9.10 × 10^–12^	*cg14535*	7	*vab-8 (2)*	–	NG2484 *vab-8 (gm84)*
rs1421085	*FTO*	Muscle–Skeletal	0.14 (C)	7.50 × 10^–08^	*–*	–	*–*	–	*–*
rs11648570	*PMFBP1*	Esophagus–Mucosa	− 0.29 (C)	9.00 × 10^–05^	*cg12702*	1	*–*	–	*–*
rs3803800	*TNFSF13*	Brain–Cortex	− 0.2 (G)	2.50 × 10^–08^	*egr*	3	*–*	–	*–*
rs1053651	*TCAP*	Muscle–Skeletal	− 0.11 (C)	4.90 × 10^–11^	*–*	–	*–*	–	*–*
rs1991556	*MAPT*	Brain–Cerebellum	− 0.28 (A)	1.80 × 10^–06^	*tau*	7	*ptl-1 (8)*	–	RB809 *ptl-1 (ok621)*
rs4815364	*ACSS1*	Brain–Cerebellar Hemisphere	− 0.47 (A)	1.70 × 10^–12^	*accoas*	6	*–*	–	*–*

*eQTL* expression quantitative trait loci.

*Alcohol SNPs associated with liver enzymes at replication P value < 0.000476. Liver enzyme GWAS summary statistics obtained from Pazoki et al. 2021.

^†^Normalized effect size and P-value obtained from GTEx portal.

^‡^Orthologs were extracted from FlyBase and WormBase. § number of FlyBase tools that support the gene-pair relationship out of a total number of tools (n = 15) that computed relationships between *Homo sapiens* and *Drosophila melanogaster*.

^¶^Genes for which mutations were homozygous lethal and as such, heterozygote mutations balanced by chromosomal translocations were instead analyzed.

#### Ortholog selection

The 24 SNPs with cis-eQTL effect with their annotated genes were matched to their orthologs in *Drosophila* using FlyBase (DIOPT online tool version 8.5/9.0; beta; http://www.flyrnai.org/diopt). To ensure the selection of the most credible orthologs, we used scores calculated in FlyBase. This database provides a number of approaches that support the gene-pair relationship out of a total number of tools that computed relationships between *Homo sapiens* and *Drosophila*. Genes with a score of > 12 were shortlisted for further analysis in *Drosophila*. Nineteen *Drosophila* orthologs were identified of which eight had a score ≥ 12 including *ARPC1B* (*arpc1*), *ACTR1B (arp1), GPN1* (*CG3704*), *WDPCP* (*frz*), *MLXIPL* (*mondo*), *SLC4A8 (ndae1), SCN8A* (*para*), and *TENM2* (*Ten-m*).

To identify potential worm orthologs, the SNPs with cis-eQTL effect with their annotated genes were sought for their worm orthologs within WormBase (https://wormbase.org/). BLASTp analysis results of human protein sequence against *C. elegans* protein database were obtained in WormBase version WS280 using data from *C. elegans* Sequencing Consortium genome project (PRJNA13758). The human protein sequence for the protein encoded by the genes under this study was extracted from UniProt (https://www.uniprot.org/). Worm genes with the best alignment with the human protein sequence indicated by an Expect value (E value) < 1 × 10^–5^ and the highest BLASTp score (bits) were moved forward. The E value represents the number of alignments that could be found in similarity to the protein sequence by chance. Using WormBase, 13 worm orthologs were identified including *ACTR1B* (*arp-*1), *ARPC1B* (*arx-3*), *GPN1* (*gop-2*), *MLXIPL* (*mml-1*), *STAT6* (*sta-1*), *TENM2* (*ten-1*), *KIF26A* (*vab-8*), *SLC4A8* (*abts-1*), *MAPT* (*ptl-1*) and *SCN8A* (cca-1). We excluded *KLB* (*klo-1*), *DRD2* (*dop-2*), and *ADH1B* (*adh-5*) from the *C. elegans* experiments as similar studies investigating these genes already exist^16–18^.

We did not identify any fly or worm orthologs for *NUCKS1*, *GCKR*, *BDNF*, *FTO*, *TCAP*. Additionally, no worm ortholog was found for *WDPCP, MSANTD1, MYBPC3, PMFBP1, TNFSF13,* and *ACSS1.*

### *Drosophila*

#### Genetics and *Drosophila melanogaster* strains

All *Drosophila* stocks and crosses were maintained on standard cornmeal agar media at 25 °C on 12/12 h light/dark cycles. The following strains were used as positive control: *w*^*berlin*^*; hppy*^*17–51*^*;* + *(hppy* mutant*)* and *w*^*berlin*^*; hppy*^*17–51*^*; hppy III* (*hppy* mutant with a rescue genomic construct) (gifts from Prof. Ulrike Heberlein, Janelia Research Campus, Virginia, USA)*,* RNAi lines were obtained from Bloomington *Drosophila* Stock Center (gene CG3704 (BDSC no: 55294), Frz (BDSC no: 55649), Mondo (BDSC no: 27059), Arpc1 (BDSC no: 31246), Para (BDSC no: 33923) and TenM (BDSC no: 29390)). All lines used were backcrossed to *w*^*1118*^ or [*v*]^*w1118*^ (RNAi lines). The expression of all RNAi constructs was driven by the ubiquitous driver, daGal4, which drives expression throughout development from embryonic to adult stage in all tissues. All the experiments on adult flies were performed using males.

#### *Drosophila* ethanol consumption assay

The CApillary FEeder (CAFE) assay^19^ was used to measure ethanol consumption. Eight male flies were placed into an experimental vial (8 cm height, 3.3 cm diameter) containing 6 microcapillary tubes (BRAND® disposable BLAUBRAND® micropipettes, intraMark, BR708707, with 1 µl marks), each containing 5 µl of liquid food. Liquid food was prepared by dissolving 50 mg of yeast granules in 1 ml of boiling water by vortexing, followed by brief centrifugation. Then, 40 mg of sucrose (Sigma‒Aldrich, 84097) was added to 800 µl of the dissolved yeast mixture, followed by vortexing. The microcapillary tubes were filled with liquid food up to the 5 µl mark. Ethanol food consists of normal food supplemented with 15% ethanol. Each experiment consisted of 5 experimental vials per genotype with each vial containing normal food (3 capillaries) and ethanol food (3 capillaries). The flies were acclimatized in the experimental vial without any food for 2 h prior to the start of the experiment. This step was also used to incentivise the flies to eat once the food was introduced. The experimental vials were placed in a plastic box with a cover to control humidity. The flies were allowed to feed for 19 h, after which the amount consumed (in mm) was measured with a digital calliper (Dasqua Bluetooth Digital Calliper 12″/300 mm, 24108120). The total amount of food consumed was calculated using the formula:

$${\text{Food}}\;{\text{uptake}}\;\left( {\mu {\text{l}}} \right) = \left( {\Sigma \;{\text{measured}}\;{\text{distances}}\;{\text{between}}\;{3}\;{\text{microcapillary}}\;{\text{tubes}}\;\left( {{\text{mm}}} \right)} \right)/{14}.{42}\;{\text{mm}}\;\left( {{1}\;\mu {\text{l}}\;{\text{in}}\;{\text{measured}}\;{\text{distance}}} \right)/{\text{8 flies}}.$$

#### *Drosophila* ethanol sedation assay

Fly sedation assay was performed as previously described^20^. Briefly, 8 flies were transferred to a 25 mm x 95 mm transparent plastic vial in between two cotton plugs. A piece of cotton plug at the base of the vial served as a stable surface to observe the flies and another plug was used to cap the vial and deliver the ethanol. 500ul of 100% ethanol was added to the side of the cotton plug facing the flies. Sedation was observed manually as ST50, which is the time in minutes it takes for 50% of the flies in a sample vial to become sedated. Sedation events are recorded when the flies become inactive and lay on their backs for over 10 s.

#### *Drosophila* triradylglycerols (TAG) measurements

To assess the role of the candidate genes in lipogenesis, we assessed the effect of RNAi knockdown of the selected genes on TAG levels in *Drosophila*. Eight male flies of the indicated genotypes were placed into an experimental vial as described in the ethanol consumption assay, with all 6 microcapillary tubes filled with normal food (5% sucrose + 5% yeast) or ethanol food (normal food + 15% ethanol), for 2 days. TAGs were assessed through colorimetric assays using 96-well microtiter plates and an Infinite M200Pro multifunction reader (TECAN). The assays were performed as previously described^21^. Briefly, flies were homogenized in 110 µl of PBS + 0.05% Tween 20 (PBST) for 2 min on ice and immediately incubated at 70 °C for 10 min to inactivate endogenous enzymatic activity. A 35 µl fly homogenate sample and a glycerol standard (Sigma, no. G7793) were incubated together with either 35 µl of PBST (for free glycerol measurements) or 35 µl of TAG reagent (Sigma, no. T2449, for TAG measurements) at 37 °C for 60 min. After 3 min of centrifugation at full speed, 30 µl of each sample was transferred into a clear-bottom plate (two technical replicates per biological sample) together with 100 µl of free glycerol reagent (Sigma‒Aldrich, F6428) and incubated at 37 °C for 5 min. TAG absorbance was divided by the protein concentration of the respective sample, which was measured by Bradford assay (Sigma‒Aldrich, B6916).

### *C. elegans*

#### Nematode strains and culture

All *C. elegans* strains were cultured on nematode growth medium (NGM) agar plates at 20 °C using *Escherichia coli* OP50 as a food source. For the wildtype worms, Bristol N2 strain was used. Genes for which mutations were homozygous lethal (*arp-1, arx-3, gop-2* and *ten-1*), heterozygote mutations balanced by chromosomal translocations were instead analyzed. Loss-of-function mutations were not backcrossed into Bristol N2, but instead initially screened for phenotypic differences with those differences validated by RNAi.

#### Nematode RNA interference (RNAi)

RNAi experiments were performed on the NL2099 *rrf-3 (pk1426)* strain as previously described^16,22^ (Supplementary Table 3). RNAi was achieved by feeding^23^ using the ORFeome based RNAi library^24^. In brief, HT115 RNAi bacterial clones were initially cultured in LB media with 100 µg/ml ampicillin and subsequently spotted in three 50 µl drops on 60 mm diameter NGM plates containing 1 mM isopropyl β-1-thiogalactopyranoside (IPTG) and 25 µg/ml carbenicillin. Plates were left to dry for 4–7 days before seeding to improve RNAi efficiency. Following seeding, five L3–L4 worms were added to each RNAi plate and cultured at 20 °C until the F1 generation reached adulthood. Ethanol experiments were performed and analyzed as described above and compared to worms fed with an empty RNAi feeding vector.

#### Nematode behavioural assays

All ethanol experiments were performed at 20 °C in a temperature-controlled room as previously described^16,22^. Behavioural assays were conducted on young adult hermaphrodites selected from sparsely populated NGM plates. Nematodes with *loss-of-function* mutations in worm orthologues of *ACTR1B* (*arp-*1), *ARPC1B* (*arx-3*), *GPN1* (*gop-2*), *MLXIPL* (*mml-1*), *STAT6* (*sta-1*), *TENM2* (*ten-1*), *KIF26A* (*vab-8*), *SLC4A8* (*abts-1*), *MAPT* (*ptl-1*) and *SCN8A* (cca-1) were acutely exposed to ethanol and the resultant effect on rate of locomotion (thrashes per minute) was quantified in Dent’s solution (140 mM NaCl, 6 mM KCl, 1 mM CaCl2, 1 mM MgCl2, 5 mM HEPES, pH 7.4 with bovine serum albumin at 0.1 mg/ml) by measuring thrashes per minute (one thrash defined as one complete movement from maximum to minimum amplitude and back) following 10 min exposure to the drug. Ethanol was mixed with Dent’s solution at a concentration of 400 mM, which has previously been shown to produce a ~ 70% reduction in locomotion rate in wild-type worms^22,25^.

### Bioinformatics

#### Secondary analysis

To gain a better insight into the biological pathways involved in the link between alcohol consumption and liver damage, we used the genetic variants within the genes highlighted by our model organism experiments and performed a series of secondary analyses using human data. We explored publicly available data from the UK Biobank deposited in the Edinburgh Gene Atlas^26^ using Phewas (Phenome-wide association analysis) databases to obtain association results between the genetic variants and 778 traits. We additionally used the genes highlighted by our model organism experiments to assess the causal effect of gene expression on liver conditions. Within these genes, the SNPs that have been identified to have a cis-eQTL effect within the previously published studies were selected and used as MR instrument against liver conditions within the *twosampleMR* package in R.

#### Statistical analyses

Within the Airwave study sample, we performed a linear regression to study the association of alcohol consumption with each of the metabolomic features (Metabolome-wide association study; MWAS). We adjusted the statistical analysis for age, sex, smoking status, and salary class. To account for multiple testing and the high degree of correlation in metabolomics datasets, we used a permutation-based method to estimate the significance level of the associations^27,28^. For each metabolomics platform, a *P*-value threshold equivalent to adjusting to a 5% Family-Wise Error Rate (i.e., Bonferroni method) was computed. A series of hypergeometric tests implemented in the R package MetaboAnalystR^29^ was used for pathway enrichment analysis where an FDR threshold of 0.05 was used as a significance threshold for each of the metabolomics platforms. To obtain an estimation for the association of known alcohol SNPs with our alcohol-associated metabolites to be used in the MR analysis, we performed linear regression analysis within the Airwave sample (see “Supplementary Methods” for details of the GWAS on metabolomics). Linear analyses between SNPs and metabolites were conducted for each metabolomic feature with adjustment for age, sex, and genetic principal components within a subsample of Airwave that included participants with both genetic and metabolite data (N = 1970). In *C. elegans*, locomotion rate was presented normalized as a percentage of the mean thrashing rate of untreated worms measured each day. All worm data were expressed as mean ± SE with an N = 30 individual worms. Locomotion rate significance was assessed by one-way analysis of variance (ANOVA) with post-hoc Tukey test for multiple comparisons. Statistical analyses of the *Drosophila* experimental data were performed using GraphPad Prism (www.graphpad.com). *Drosophila* data were presented as the mean values, and the error bars indicate ± SD.

## Results

To understand biological effects of alcohol consumption in human population, we investigated the circulating metabolites within the Airwave study sample using an agnostic approach which revealed the association of 152 unique metabolites with alcohol consumption (Supplementary Data). Using MR approach to examine the causality of the above associations, we identified a possible causal association (Table 2) of alcohol consumption on changing circulating level for several lipid metabolites (TAGs, Diradylglycerols, Glycerophosphocholines, Sphingolipids), and an alkaloid (piperine). The most statistically significant causal association was observed with a triacylglycerol TG 60:2 (β = 1.24; 95% CI 0.52,1.95; *P*-value = 0.002). Pathway analysis on the 152 unique alcohol-metabolite associations showed that the linoleic acid (LNA) and alpha linolenic acid (ALA) metabolism pathway (LNA/ALA) within the Small Molecule Pathway Database (SMPDB) was enriched with alcohol-associated metabolomic features (Pfdr = 5.67 × 10^–3^). These features were annotated to Tetracosapentaenoic acid (24:5n-3; β = 0.01; 95% CI 0.008,0.012; *P*-value = 4.2 × 10^–12^), Eicosapentaenoic acid (β = 0.009; 95% CI 0.007,0.011; *P*-value = 2.4 × 10^–12^), Stearidonic acid (β = 0.008; 95% CI 0.006,0.01; *P*-value = 2.2 × 10^–9^), Arachidonic acid (β = 0.007; 95% CI 0.005,0.009; *P*-value = 9.7 × 10^–8^) and Adrenic acid (β = 0.01; 95% CI 0.008,0.012; *P*-value = 9.7 × 10^–8^).

**Table 2:** Overview of the causal effect of alcohol on circulating metabolites using the inverse variance weighted two-sample Mendelian randomization multiple instrument method.

Metabolite	Name	Main class	Beta (95% CI)	P-value
SLPOS_457.3339_0.7190	CAR DC18:1	Fatty esters	-1.13 (-2.14, -0.11)	0.036
SLPOS_579.5352_8.2395	DG 34:0	DAG	0.91 (0.05,1.77)	0.047
SLPOS_606.5548_8.3326	DG 36:1	DAG	0.92 (0.07,1.77)	0.041
SLPOS_745.5603_5.7424	PC 33:2	Glycerophosphocholines	0.78 (0.07,1.5)	0.040
SLPOS_629.5424_6.9217	PE 38:4	Glycerophosphoethanolamines	1.2 (0.15,2.25)	0.032
SHPOS_625.5174_2.8914	PE 38:5	Glycerophosphoethanolamines	1.1 (0.1,2.1)	0.039
SLPOS_201.0516_0.6580	Piperine	Alkaloids	1.16 (0.1,2.22)	0.040
SHPOS_807.6348_4.4974	SM 40:2;O2	Sphingomyelins	1.06 (0.15,1.96)	0.028
SHPOS_827.7093_0.5837	TG 48:1	TAG	0.95 (0.13,1.77)	0.030
SLPOS_898.7965_11.2257	TG 53:1	TAG	0.93 (0.1,1.75)	0.036
SLPOS_888.8079_10.7574	TG 53:3	TAG	0.96 (0.06,1.85)	0.045
SLPOS_607.5599_11.1277	TG 54:2	TAG	0.95 (0.19,1.71)	0.019
SLPOS_1027.7392_10.9051	TG 54:3	TAG	1.06 (0.21,1.91)	0.020
SLPOS_984.8508_11.7812	TG 58:1	TAG	1.12 (0.44,1.81)	0.003
SLPOS_687.6307_11.5996	TG 58:2	TAG	1.08 (0.27,1.9)	0.014
SLPOS_689.6461_11.7895	TG 60:2	TAG	1.24 (0.52,1.95)	0.002

Effect estimates and 95% CI is given for the inverse variance weighted method of Mendelian randomization.

*CI* confidence interval, *DAG* diradylglycerols, *TAG* triradylglycerols.

**Figure 2:**
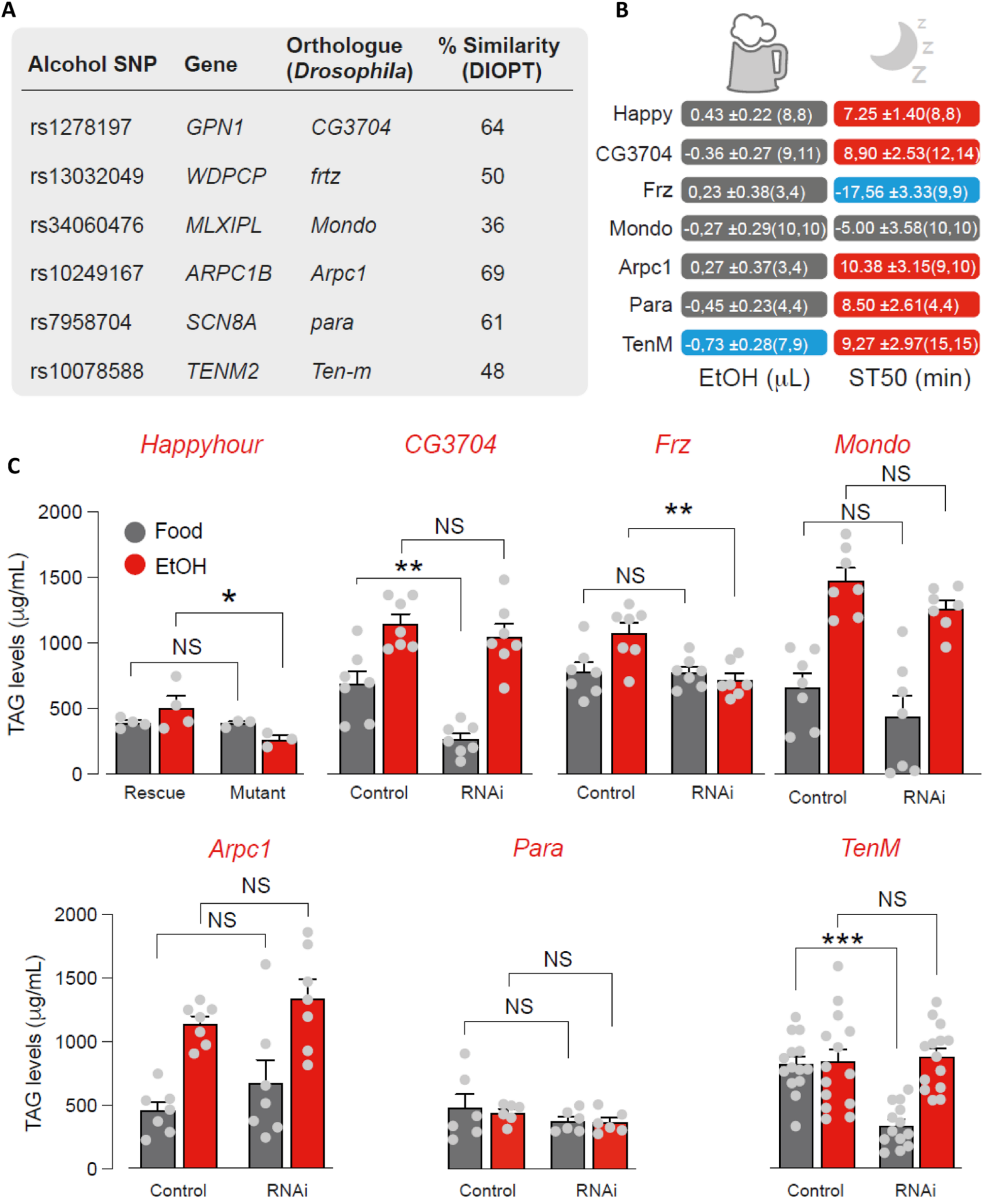
Analysis of alcohol intake and sedation in adult flies. (**A**) Mapping *Drosophila* orthologues of human genes involved in ethanol consumption. Similarity of protein alignment based on the DRSC integrative ortholog prediction tool (DIOPT). (**B**) Analysis of ethanol intake (left column) and sedation (right column) in the *Drosophila* RNAi lines. The numerical values show the difference between means ± standard error of mean (RNAi–Control). Red or blue correspond to the measurements that were significantly increased or decreased, respectively (P ≤ 0.05) whereas grey indicates a significance higher than 0.05. The statistical significance was determined using an unpaired t-test. Values in parenthesis are the number of biological replicates for respectively, the control and the RNAi line. (**C**) Analysis of TAG levels in adult flies fed either with normal or ethanol-containing food (mean ± standard error of mean; asterisks, 2-way ANOVA with Tukey's multiple comparisons test). The number of biological replicates per experimental variable (n) is indicated in either the respective figure or figure legend. No sample was excluded from the analysis unless otherwise stated. Blinding was not performed. Normality was assessed before deciding on which parametric or non-parametric test to use for inferential statistics. Statistical significance is indicated as * for P < 0.05, ** for P < 0.01, *** for P < 0.001, **** for P < 0.0001 and NS for P ≥ 0.05.

We used *Drosophila hppy* mutant, described to have an increased resistance to ethanol sedation^30^, as positive control in all fly experiments. *Drosophila* with knock-down of *ARPC1B* (*arpc1*), *GPN1* (*CG3704*), *WDPCP* (*frz*), *MLXIPL* (*mondo*), *SCN8A* (*para*), and *TENM2* (*Ten-m*) together with *hppy* mutant were exposed to food supplemented with 15% Ethanol in a CAFE assay. A significant difference (Fig. 2A,B) was observed between ethanol consumed (μL) by flies with RNAi knockdown of *TENM2* (*Ten-m*). Following exposure to ethanol vapour, the effect on Sedation Time 50% (ST50; minute) was quantified and we observed that in comparison to control (*hppy*), RNAi knockdown of fly orthologues of *WDPCP* (*frz*) showed a faster rate of sedation whilst *TENM2* (*Ten-m*), *GPN1* (*CG3704*), *ARPC1B* (*arpc1*) and *SCN8A* (*para*) showed a slower rate of sedation indicated by a higher ST50. *WDPCP* (frz), *TENM2* (Ten-m) and *GPN1* (CG3704) knockdown flies showed reduced TAG levels (Fig. 2C).

**Figure 3:**
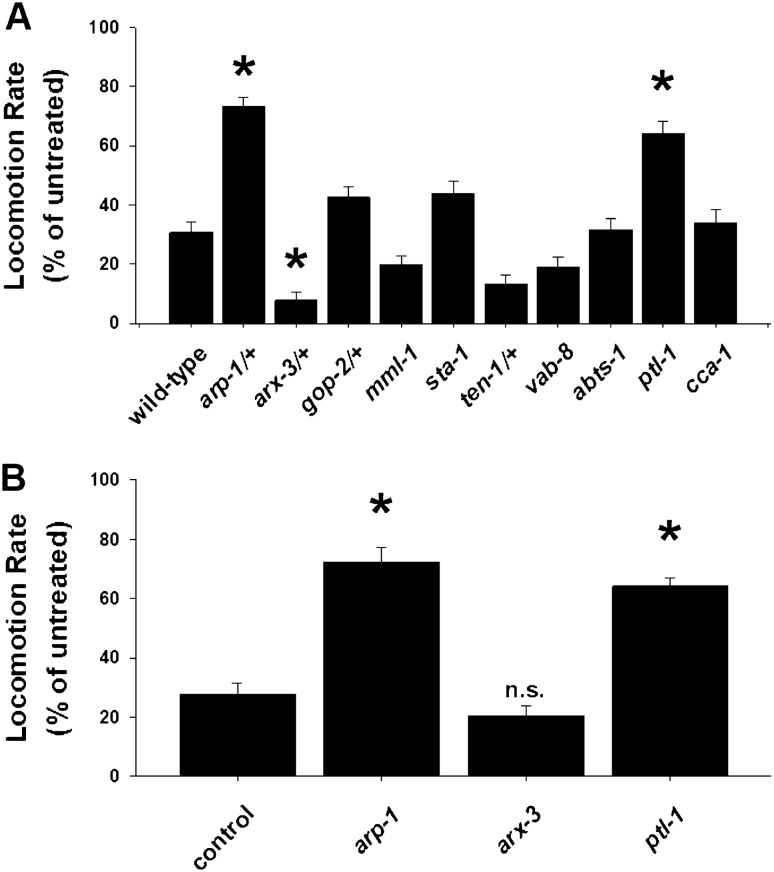
Quantification of alcohol phenotypes for *C. elegans* genes. (**A**) Nematodes with *loss-of-function* mutations in worm orthologues of *ACTR1B* (*arp-*1/ +), *ARPC1B* (*arx-3/* +), *GPN1* (*gop-2/* +), *MLXIPL* (*mml-1*), *STAT6* (*sta-1*), *TENM2* (*ten-1/* +), *KIF26A* (*vab-8*), *SLC4A8* (*abts-1*), *MAPT* (*ptl-1*) and *SCN8A* (cca-1) were acutely exposed to ethanol and the resultant effect on rate of locomotion (thrashes per minute) was quantified. In comparison with Bristol N2 wild-type worms, significant differences were identified for *arp-1*, *arx-3* and *ptl-1*. Data is presented normalized to locomotion rate of untreated worms. *P < 0.01. (B) RNAi confirmation of positively identified genes involved in alcohol phenotypes. In comparison to controls, RNAi knockdown of worm orthologues of *ACTR1B* (*arp-*1) and *MAPT* (*ptl-1*) phenocopied the *loss-of-function* mutations, whereas *ARPC1B* (*arx-3*) knockdown had no effect. *P < 0.01. n.s., not significant.

*C. elegans loss-of-function* mutants of *TENM2* (*ten-1)*, *KIF26A (vab-8)*, *SLC4A8 (abts-1)* and *SCN8A (cca-1)* showed significant differences in basal locomotion rate (Supplementary Fig. S1A). After acute exposure to ethanol in *C. elegance loss-of-function* mutants, significant differences were identified in normalized locomotion rate of *ACTR1B* (*arp-*1), *ARPC1B* (*arx-3*) and *MAPT* (*ptl-1*) in comparison with Bristol N2 wild-type worms (Fig. 3A). RNAi knockdown of these genes confirmed that in comparison to controls, RNAi knockdown of worm orthologues of *ACTR1B* (*arp-*1) and *MAPT* (*ptl-1*) and *ARPC1B* (*arx-3*) did not have any effect on basal locomotion rate (Supplementary Fig. S1B) but RNAi knockdown of *ACTR1B* (*arp-*1) and *MAPT* (*ptl-1*) phenocopied the *loss-of-function* mutations after exposure to ethanol (Fig. 3B).

### Secondary analysis

The Phewas analysis (Table 3) on the three genetic variants within *WDPCP*, *TENM2*, and *GPN1* showed a link between rs10078588 (*TENM2*) and food and liquid intake (beef, oily fish, fresh fruit, bread, alcohol). rs13032049 (*WDPCP*) was linked to salt intake and smoking. rs2178197 (*GPN1*) was linked to hypertension, and hematologic traits. All three SNPs showed association with disorders of lipoprotein metabolism and other lipidaemias (ICD code E78) within the UK Biobank (Table 4). We finally performed a Mendelian randomization analysis on the expression of *WDPCP* gene (ENSG00000115507) and liver conditions using cis-eQTL data (Table 5) and identified a link between expression of *WDPCP* with liver fibrosis and cirrhosis (β = − 0.20; 95% CI − 0.39, − 0.01; *P*-value = 0.04) as well as liver and bile duct cancer (β = 0.0003; 95% CI 3.27 × 10^–05^, 5.85 × 10^–04^; *P*-value = 0.02). We also observed a suggestive link with the changes in liver fatty acid-binding protein (Table 5).

**Table 3:** Overview of the significant associations between SNPs in *WDPCP*, *TENM2*, and *GPN1* with Phewas traits within the UK Biobank Edinburgh Gene Atlas*.*

Trait	Beta	Z value	P-value
rs10078588-A (*TENM2*)			
Bread intake, number of slices/week	**0.09**	**5.66**	**7.57 × 10**^**–9**^
Alcohol weekly intake frequency (high to low frequency)	**− 0.02**	**5.21**	**9.63 × 10**^**–8**^
Oily fish intake, number of times/week	**− 0.01**	**4.53**	**2.88 × 10**^**–6**^
Beef intake, number of intake times/week	**0.01**	**4.45**	**4.34 × 10**^**–6**^
Fresh fruit intake, number of pieces/day	**− 0.01**	**4.44**	**4.45 × 10**^**–6**^
rs13032049-G (*WDPCP*)			
Salt added to food, frequency	**0.01**	**5.01**	**2.77 × 10**^**–7**^
Smoking status, current *vs.* previous and never	**0.01**	**4.12**	**1.88 × 10**^**–5**^
rs2178197-G (*GPN1*)			
Hypertension	**− 3.60 × 10**^**–3**^	**− 4.26**	**1.02 × 10**^**–5**^
Mean sphered cell volume, fl	**0.04**	**− 4.98**	**3.15 × 10**^**–7**^
Red blood cell (erythrocyte) count, × 10^12^ cells/L	**− 3.30 × 10**^**–3**^	**− 5.41**	**3.21 × 10**^**–8**^
Number of treatments/medications taken	**− 0.02**	**− 4.64**	**1.71 × 10**^**–6**^
Monocyte percentage, %	**0.02**	**− 4.97**	**3.39 × 10**^**–7**^
Number of operations, self-reported	**− 0.01**	**− 4.33**	**7.58 × 10**^**–6**^
Mean reticulocyte volume, fL	**0.06**	**− 4.39**	**5.66 × 10**^**–6**^
High light scatter reticulocyte percentage, %	**− 1.60 × 10**^**–3**^	**− 4.37**	**6.32 × 10**^**–6**^
Reticulocyte count, × 10^12^ cells/L	**− 2.00 × 10**^**–4**^	**− 5.15**	**1.33 × 10**^**–7**^
Reticulocyte percentage, %	**− 4.20 × 10**^**–3**^	**− 4.09**	**2.12 × 10**^**–5**^
Lymphocyte percentage, %	**0.06**	**− 4.49**	**3.59 × 10**^**–6**^
Neutrophil percentage, %	**− 0.07**	**− 4.48**	**3.81 × 10**^**–6**^
High light scatter reticulocyte count, × 10^12^ cells/L	**− 1.00 × 10**^**–4**^	**− 5.39**	**3.50 × 10**^**–8**^

Significant associations are depicted in bold. Statistical significance level *P*-value = 2.14 × 10^–5^ was considered equivalent to a *P*-value adjusted for multiple testing for the analysis of 3 SNPs and 778 phenotypes within the Gene Atlas. The associations are provided per copy of the allele that increases alcohol intake.

**Table 4:** Overview of the association of SNPs in *WDPCP*, *TENM2*, and *GPN1* with disorders of lipoprotein metabolism and other lipidaemias (ICD code E78) within the UK Biobank Edinburgh Gene Atlas.

SNP	chromosome	Base pair location	Effect allele	Beta (effect estimate)	95% CI lower bound	95% CI upper bound	P-value	MAF	HWE
rs10078588	5 (*TENM2)*	166,816,176	A	− 0.001	− 2.49 × 10^–04^	− 2.44 × 10^–03^	0.02	0.47	0.5
rs13032049	2 (*WDPCP*)	63,581,507	G	− 0.001	− 1.59^–04^	− 2.60 × 10^–03^	0.03	0.28	0.51
rs2178197	2 (*GPN1*)	27,860,551	G	− 0.001	− 3.36 × 10^–04^	− 2.55 × 10^–03^	0.01	0.42	0.21

Results obtained from the Edinburgh Gene Atlas.

*CI* confidence interval, *SNP* single nucleotide polymorphism, *MAF* minor allele frequency, *HWE* Hardy Weinberg Equilibrium.

**Table 5:** Overview of the significant results from inverse variance weighted Mendelian randomization analysis for the effect of gene expression of ENSG00000115507 on liver traits using the MRC IEU OpenGWAS data infrastructure^51^.

Liver trait	Number of SNPs	Effect estimate	Standard error	*P* value
Fatty acid-binding protein, liver	6	− 0.10	0.05	0.06
Liver enzyme levels (alanine transaminase)	6	− 0.002	0.001	0.2
Fibrosis and cirrhosis of liver	6	− 0.20	0.1	0.04
Liver & bile duct cancer	6	0.0003	0.0001	0.03

## Discussion

In this study we used data from humans, *C. elegans*, and *Drosophila* and identified a link between genes implicated in alcohol consumption and lipid metabolism. We identified that alcohol consumption changes the metabolites within linoleic acid (LNA) and alpha linolenic acid (ALA) metabolism pathway (LNA/ALA) and demonstrates causal effect on changes in several lipid metabolites. We highlighted that change of function of the genes implicated in alcohol consumption leads to changes in ethanol consumption, sedation after exposure to ethanol vapor, and changes in accumulation of fat in *Drosophila* as well as changes in locomotion rate after exposure to ethanol in *C. elegans.* Our results demonstrate that three alcohol-implicated genes namely *WDPCP* (*frz*), *TENM2* (*Ten-m*), and *GPN1* (*CG3704*) might be involved in fat accumulation.

In our study, we identified several metabolites to be causally altered due to alcohol consumption and identified the LNA/ALA pathway involved in alcohol consumption. A metabolite, eicosapentaenoic acid that is synthesized from linolenic acid, has previously been associated with altered acute behavioral responses to alcohol in *C. elegans* (altered locomotion)^31^, and mice^32^. In addition in humans, long-chain polyunsaturated fatty acids are shown to be associated with alcohol sensitivity^33^ and genes that are essential to generate ω-3 long-chain polyunsaturated fatty acids are shown to be associated with alcohol-related phenotypes^34^. Previous studies also showed associations between lipid metabolites and alcohol consumption^35^. We previously showed alteration of high-density lipoprotein to be associated with alcohol consumption within the UK Biobank^10^. The metabolomics part of the current study is a step forward in that firstly it uses causal inference tools (MR method) to demonstrate the causality of the association between lipid metabolites specifically long chain fatty acids with alcohol consumption. Secondly, by performing a pathway analysis, we were able to pinpoint a specific LNA/ALA pathway in alcohol consumption.

In our study, we used alcohol-associated genetic variants to explore (1) the alcohol-induced biological changes in human metabolites and (2) alcohol-induced biological effect of genes annotated to alcohol-associated genetic variants in *C. elegans* and *Drosophila melanogaster*. Of the alcohol-implicated genes that we investigated in *C. elegans*, *ACTR1B* (*arp-*1) and *MAPT* (*ptl-1*), show significant effects on the worms’ locomotion upon acute exposure to ethanol. In addition, *TENM2* (*Ten-m*) shows significant effects on ethanol consumption in *Drosophila* and apart from *MLXIPL* (*mondo*), RNAi lines for all the genes investigated in *Drosophila* change the time to sedation from ethanol.

*WDPCP* which was first characterized in *Drosophila*^36^ is known to be involved in cell polarity and ciliogenesis^37,38^. In humans, mutations in *WDPCP* gene cause Bardet-Biedl Syndrome^39^ presenting with a variety of symptoms in different organs including obesity, blindness, and polydactyly. *WDPCP* gene is widely expressed known to be involved in hedgehog signaling^40^. Recently, Liu and colleagues^13^ identified that a G allele in rs13032049 within the *WDPCP* gene was associated with an increased consumption of alcohol. The G allele in rs13032049 shows a strong association with a lower expression of the *WDPCP* gene. We found the SNP to be strongly associated with increased liver enzyme GGT and behavioral traits such as smoking and adding salt to food. In our Drosophila experiments, the observed effect of *WDPCP* (*frz*) knockdown on the changed TAG levels occurred under exposure to ethanol and followed similar patterns as *hppy* mutants. This implies that minimizing alcohol consumption could reduce fat accumulation and thus could potentially reduce the risk of hepatic lipogenesis. In our further MR analysis, we used publicly available databases derived from GWAS in human population and showed a link between the gene expression of *WDPCP* and liver fibrosis and liver cirrhosis. The analysis also showed a suggestive link with the liver fatty acid binding protein that is involved in the metabolism of lipids^41^. This evidence suggests that *WDPCP* might be an important gene involved in the pathway between alcohol consumption, accumulation of fat and liver fibrosis. This could have public health implications in terms of the identification of high-risk groups and targeting preventive measures as well as drug development. More studies in vivo and in vitro are needed to focus on *WDPCP* and provide more details on its role in lipid metabolism and liver pathologies.

Changes in TAG levels of *Drosophila* occurred with exposure to normal food rather than ethanol for RNAi knockdown of *TENM2* (*Ten-m*) and *GPN1* (*CG3704*) indicating that loss of function of these genes could have a direct role in the accumulation of fat in liver independent of exposure to ethanol. RNAi knockdown of both genes shows increased tolerance to the sedative effect of ethanol which could justify the effect of these genes on a more frequent alcohol consumption in humans, possibly due to alcohol tolerance. *GPN1* is located on chromosome 2p23.3 and the encoded protein is implicated in the regulation of TGFβ superfamily signaling^42^ that is demonstrated to play a role in obesity^43^, accumulation of fat in the liver^42,44^ as well as regulation of *ADH1* gene that enhances alcohol-induced liver damage and lipid metabolism^45^. The existing evidence alongside our findings on the role of *GPN1* in alcohol consumption and lipid metabolism in *Drosophila* implies that *GPN1* might play a role upstream of TGFβ in the regulation of the metabolism of alcohol and lipids. Further studies are needed to highlight the relationship between *GPN1* and TGFβ in alcohol consumption and alcohol-induced liver damage.

*TENM2* (*Ten-m*) is located on chromosome 5q34, and the encoded protein is involved in cell adhesion^46^. *TENM2* is found to be highly enriched in white adipocyte progenitor cells^47^. *TENM2* deficiency in human fat cells leads to expression of UCP1, the primary marker of brown adipose tissue^48^. Genetic variants in *TENM2* have shown to be linked to obesity^49^. Our secondary analyses confirmed an association between the genetic variant in *TENM2* and excess of food and liquid intake which suggests the link between *TENM2,* and alcohol consumption could also be due to systematic increase in consumption of all food and beverages rather than alcohol alone. The evidence in this study alongside the existing literature highlights that the observed effect of *TENM2* (*Ten-m*) RNAi knockdown on the changes in TAG levels in *Drosophila* could potentially be related to biological pathways implicated in adipose tissue rather than pure liver-related pathways.

One strength of our study is in that we performed our analyses in human and two different model organisms, allowing for a more comprehensive insight into biological mechanisms involved in the function of alcohol consumption genes under different biological scenarios. A second strength of this study is in the use of RNAi technique which provides insight into the function of genes and what biological manifestation they would have when exposed to ethanol. The third strength of our study is in the use of CAFE assay that allows for the investigation of food and alcohol consumption in *Drosophila* in a more controlled environment. In our CAFE assay, each experimental box per genotype contained both normal food and ethanol food (food supplemented with 15% ethanol) providing the insects with a choice. Another strength of our study is that in our TAG levels experiments, we made our conclusions based on the comparisons between flies (RNAi vs. control) that were exposed to identical food and environmental conditions which increases the robustness of our conclusions. Finally, we combined the results with human studies to get better insight into the link between alcohol consumption and lipid metabolites.

Alcohol consumption in Airwave participants was calculated based on self-reported data which could have affected the precision of the alcohol consumed due to recall bias. To reduce this limitation, the duration of recall was limited to the last 7 days in the Airwave study. We should acknowledge that the concentration of alcohol within alcoholic drinks is not standard^50^, and our calculation of alcohol consumed could be affected by these variations. Although the genetic variants used for our investigations were originally found in human studies and we also performed a metabolomics analysis between alcohol consumption and circulating metabolites in the human population, the main part of the study was performed in model organisms and the results of this study might not directly generalisable to patients and the public without performing further population studies.

## Conclusion

We found that alcohol-associated genes may be involved in the metabolism of lipids. Our study highlights three genes (*WDPCP, TENM2,* and *GPN1*) that may be involved in the accumulation of lipids. Of these genes, *WDPCP* exhibits its effects on lipid accumulation in *Drosophila* with exposure to ethanol. The gene expression of *WDPCP* in the human population supports a link to liver fibrosis. Further studies are necessary to investigate the role of this gene in ALD.

### Supplementary Information

Supplementary Information.

## Data Availability

All data generated or analyzed during this study are included in this published article (and its Supplementary Information files).
