# Supplementary material for: Evidence for involvement of the alcohol consumption WDPCP gene in lipid metabolism, and liver cirrhosis
Source: Sci Rep. 2023 Nov 23;13:20616. doi: 10.1038/s41598-023-47371-7 (PMC10667215; doi:10.1038/s41598-023-47371-7)
Supplement: Supplementary file 1 — Supplementary Information. [file 41598_2023_47371_MOESM1_ESM.docx]

Supplementary information for

Evidence for Involvement of *WDPCP* Gene in Alcohol Consumption, Lipid Metabolism, and Liver Cirrhosis

Felix O’Farrell ^1*^, MSc; Benjamin Aleyakpo^2*^, MSc, PhD; Rima Mustafa ^3,4*^, MD, PhD; Xiyun Jiang ^1*^, MSc; Rui Climaco Pinto^3, 5*^, PhD; Paul Elliott ^3-8^, MB, PhD; Ioanna Tzoulaki ^3,9^, PhD; Abbas Dehghan ^3^, MD, PhD; Samantha H. Y. Loh^10^, PhD; Jeff W. Barclay^11^, PhD; L. Miguel Martins^10^, PhD; Raha Pazoki ^1,3^, MD, PhD

1. Cardiovascular and Metabolic Research Group, Division of Biosciences, Department of Life Sciences, College of Health and Life Sciences, Brunel University, London, UB8 3PH, United Kingdom.

2. The Francis Crick Institute, London, NW1 1AT, United Kingdom.

3. School of Public Health, St Mary’s campus, Norfolk Place, London W2 1PG, United Kingdom.

4. UK Dementia Research Institute, Imperial College London, Exhibition Road, London, SW7 2AZ, United Kingdom.

5. MRC Centre for Environment and Health, Department of Epidemiology and Biostatistics, School of Public Health, St Mary’s campus, Norfolk Place, London W2 1PG, United Kingdom.

6. British Heart Foundation Centre of Research Excellence, Imperial College London, Du Cane Road, W12 0NN, United Kingdom.

7. National Institute for Health Research, Imperial Biomedical Research Centre, Imperial College London, Exhibition Road, London, SW7 2AZ, United Kingdom.

8. Health Data Research UK at Imperial College London, Exhibition Road, London, SW7 2AZ, United Kingdom.

9. Centre for Systems Biology, Biomedical Research Foundation, Academy of Athens, Athens, Greece.

10. MRC Toxicology Unit, University of Cambridge, Gleeson Building, Tennis Court Road, Cambridge CB2 1QR, UK.

11. Department of Molecular Physiology and Cell Signalling, Institute of Systems, Molecular and Integrative Biology, University of Liverpool, Liverpool L69 3BX.

# Supplementary Methods

*Study Population.* The Airwave Health Monitoring Study (the Airwave Study) was first established in 2004 as a large-scale occupational cohort of police officers. A total of 53,114 participants were enrolled by the end of baseline recruitment in March 2015. Initially, it aimed to investigate the health outcomes related to the use of Terrestrial Trunked Radio (TETRA). This cohort has also been expanded to investigate the health of workforces in more general. The rationale, design, and methods of this study can be found elsewhere [1]. The Airwave Health Monitoring Study was approved by the National Health Service Multi-site Research Ethics Committee (MREC/13/NW/0588).

*Metabolomic assays.* A random sample of 2063 participants in the Airwave study were assayed for various metabolites. Blood samples were collected at the screening visit (with informed consent) and stored at -80 °C, before being transferred to a biorepository facility and stored in vapor phase liquid nitrogen. The metabolomic assays were performed using Liquid chromatography-mass spectrometry (LC-MS) at the Imperial Phenome Centre (London, UK). Lithium heparin was utilized as an anticoagulant for the LC-MS plasma samples. Metabolomics data was acquired using Ultra Performance Liquid Chromatography-Mass Spectrometry (UPLC-MS) and Quality Control (QC) methods that were previously described [2-4]. This involved preparing a pooled study reference sample for each population and regular analysis of QC samples throughout data acquisition. Additionally, mixtures of authentic reference standards were added to the study reference, long-term reference, and study samples used in the UPLC-MS analysis to enable targeted monitoring of data quality during acquisition. Plasma samples were prepared and underwent UPLC-MS profiling analysis for lipids and small metabolites as previously described by Izzi-Engbeaya and colleagues [3]. The only deviation from the methodology in Izzi-Engbeaya and colleagues was that 100μl of samples was used without dilution prior to addition of isopropanol for the lipidomics analyses. Batches of 80 samples were prepared into 96-well plates. Each sample was mixed with four parts of 4°C isopropanol, incubated at 4°C, centrifuged, and the supernatant aliquoted into a 96-well plate. All analyses were acquired on Acquity UPLC systems coupled to Xevo G2-S ToF mass spectrometers (Waters Corporation, Milford, Massachusetts, United States). Three platforms were used for these samples: reverse phase in positive ionization mode which detects largely lipid species (Lipid Positive mode, LPOS), reverse phase in negative ionization mode which detects largely lipid species (Lipid Negative mode - LNEG) and Hydrophilic Interaction Chromatography (HILIC) in positive mode which detects largely small and polar metabolites (HILIC Positive mode, HPOS).

*Metabolomics Data processing.* Peak picking was performed using Bioconductor R-package XCMS [5]. Drift correction was achieved using a previously described method [6]. Then, we replaced negative values with zeros, and added one before log-transforming the data. Metabolomic features were filtered based on retention time to exclude non-retained and cleaning phase features - only features in the following retention times (in minutes) were accepted: HPOS (0.5-7); LNEG (0.3-9.5); LPOS (0.45-12). Principal components analysis (PCA) was used to identify outlier samples, which were then excluded. Furthermore, we excluded single-point values that were more than 5 Median Absolute Deviations (MAD) from the median. We used 10 principal components from genome-wide scans to adjust for population stratification. Finally, we transformed the data into z-scores using median and MAD so that we had comparable intensities across studies.

*UPLC-MS metabolite annotation.* Lipid annotation was initially completed by matching accurate mass fragmentation measurements to reference spectra from online databases (LIPID MAPS, Metlin, HMDB) and previous publications. Where chemical reference materials were commercially available (Avanti Polar Lipids, Sigma Aldrich, Cayman Scientific), they were used to generate definitive molecular identification by direct matching of chromatographic and spectral qualities (including accurate mass, MS/MS spectra, and isotopic distribution) to those observed in the profiling data.

**References**

1. Elliott, P.; Vergnaud, A.C.; Singh, D.; Neasham, D.; Spear, J. Heard, A., The Airwave Health Monitoring Study of police officers and staff in Great Britain: rationale, design and methods*.* *Environ Res*, **2014**. *134*, 280-5.

2. Lewis, M.R.; Pearce, J.T.M.; Spagou, K.; Green, M.; Dona, A.C.; Yuen, A.H.Y.; David, M.; Berry, D.J.; Chappell, K.; Horneffer-Van Der Sluis, V.; et al., Development and Application of Ultra-Performance Liquid Chromatography-TOF MS for Precision Large Scale Urinary Metabolic Phenotyping*.* *Analytical Chemistry*, **2016**. *88*, 9004-9013.

3. Izzi-Engbeaya, C.; Comninos, A.N.; Clarke, S.A.; Jomard, A.; Yang, L.; Jones, S.; Abbara, A.; Narayanaswamy, S.; Eng, P.C.; Papadopoulou, D.; et al., The effects of kisspeptin on β-cell function, serum metabolites and appetite in humans*.* *Diabetes, obesity & metabolism*, **2018**. *20*, 2800-2810.

4. Dona, A.C.; Jiménez, B.; Schäfer, H.; Humpfer, E.; Spraul, M.; Lewis, M.R.; Pearce, J.T.M.; Holmes, E.; Lindon, J.C. Nicholson, J.K., Precision High-Throughput Proton NMR Spectroscopy of Human Urine, Serum, and Plasma for Large-Scale Metabolic Phenotyping*.* *Analytical Chemistry*, **2014**. *86*, 9887-9894.

5. Smith, C.A.; Want, E.J.; O'maille, G.; Abagyan, R. Siuzdak, G., XCMS:  Processing Mass Spectrometry Data for Metabolite Profiling Using Nonlinear Peak Alignment, Matching, and Identification*.* *Analytical Chemistry*, **2006**. *78*, 779-787.

6. Dunn, W.B.; Broadhurst, D.; Begley, P.; Zelena, E.; Francis-Mcintyre, S.; Anderson, N.; Brown, M.; Knowles, J.D.; Halsall, A.; Haselden, J.N.; et al., Procedures for large-scale metabolic profiling of serum and plasma using gas chromatography and liquid chromatography coupled to mass spectrometry*.* *Nature Protocols*, **2011**. *6*, 1060-1083.

7. Hastie T, T.R., Narasimhan B, Chu G, *impute: Imputation for microarray data.* 2020.

8. Roshchupkin, G.V.; Adams, H.H.H.; Vernooij, M.W.; Hofman, A.; Van Duijn, C.M.; Ikram, M.A. Niessen, W.J., HASE: Framework for efficient high-dimensional association analyses*.* *Scientific Reports*, **2016**. *6*, 36076.

**Supplementary Table 1- Characteristics of the Airwave sample in which the genetic analysis for the candidate metabolites (alcohol-associated) were performed.**

| **Characteristics** | **The Airwave Study** |
| --- | --- |
|  |  |
| Number of participants | 1,970 |
| Male (%) | 1175 (59.64) |
| Age (years), mean ± SD | 40.84 ± 9.08 |
| Body mass index (kg/m2), mean ± SD | 27.05 ± 4.29 |
| Total cholesterol (mmol/L), mean ± SD | 5.25 ± 0.99 |
| HDL (mmol/L), mean ± SD | 1.51 ± 0.39 |
| SBP (mmHg), mean ± SD | 130.87 ± 15.34 |
| DBP (mmHg), mean ± SD | 79.54 ± 9.91 |
| Current smoker (%) | 169 (8.58) |
| Highest level of education completed |  |
| A levels / Highers or equivalent (NVQ3) | 616 (31.27) |
| Bachelor Degree or equivalent (NVQ4) | 382 (19.39) |
| GSCE/O-Level/CSE | 623 (31.62) |
| Left school before taking O levels / GCSEs | 82 (4.16) |
| Postgraduate qualifications | 139 (7.06) |
| Vocational qualifications (NVQ1+2) | 128 (6.50) |

**Supplementary Table 2- Overview of the test statistics for the association of 105 alcohol SNPs with liver enzymes within the UK Biobank.**

| SNP | Beta estimate (ALT) | Standard error (ALT) | P- value(ALT) | Beta estimate (ALP) | Standard error (ALP) | P (ALP) | Beta estimate (GGT) | Standard error (GGT) | P (GGT) | Liver enzyme the SNP is associated with |
| --- | --- | --- | --- | --- | --- | --- | --- | --- | --- | --- |
| rs10004020 | 0.0001 | 4.32 ×10-4 | 0.9 | 0.0004 | 2.57 ×10-4 | 0.11 | 0.0003 | 5.71 ×10-4 | 0.62 |  |
| rs10028756 | 0.0012 | 5.74 ×10-4 | 0.047 | 0.0021 | 3.42 ×10-4 | 3.90 ×10-9 | 0.0023 | 7.59 ×10-4 | 0.0035 | ALP |
| rs10078588 | 0.0003 | 3.90 ×10-4 | 0.5 | 0.0003 | 2.33 ×10-4 | 0.24 | 0.0027 | 5.16 ×10-4 | 1.30 ×10-7 | GGT |
| rs10085696 | 0.0011 | 5.00 ×10-4 | 0.024 | 0.0002 | 2.98 ×10-4 | 0.7 | 0.0004 | 6.62 ×10-4 | 0.52 |  |
| rs10249167 | 0.0005 | 5.87 ×10-4 | 0.36 | 0.0005 | 3.50 ×10-4 | 0.15 | 0.0032 | 7.77 ×10-4 | 1.70 ×10-5 | GGT |
| rs10438820 | 0.0003 | 4.23 ×10-4 | 0.56 | 0.0001 | 2.52 ×10-4 | 0.79 | 0.0008 | 5.60 ×10-4 | 0.2 |  |
| rs10496076 | 0.0005 | 4.08 ×10-4 | 0.23 | 0.0002 | 2.43 ×10-4 | 0.51 | 0.0012 | 5.39 ×10-4 | 0.042 |  |
| rs10506274 | 0.0002 | 3.89 ×10-4 | 0.57 | 0.0001 | 2.32 ×10-4 | 0.64 | 0.0016 | 5.13 ×10-4 | 0.0032 |  |
| rs1053651 | 0.0013 | 4.40 ×10-4 | 0.0048 | 0.0032 | 2.62 ×10-4 |  | 0.0022 | 5.83 ×10-4 | 0.00034 | GGT; ALP |
| rs10753661 | 0.0001 | 4.18 ×10-4 | 0.87 | 0.0001 | 2.46 ×10-4 | 0.89 | 0.0012 | 5.52 ×10-4 | 0.024 |  |
| rs10876188 | 0.0001 | 3.90 ×10-4 | 0.85 | 0.0009 | 2.32 ×10-4 | 0.00021 | 0.0034 | 5.15 ×10-4 | 1.30×10^-^10 | ALP; GGT |
| rs1104608 | 0.0008 | 3.97 ×10-4 | 0.028 | 0.0005 | 2.37 ×10-4 | 0.039 | 0.0011 | 5.26 ×10-4 | 0.033 |  |
| rs112635299 | 0.0213 | 1.37 ×10-3 |  | 0.0134 | 8.18 ×10-4 |  | 0.0114 | 1.81 ×10-3 | 3.20×10^-^11 | GGT; ALP; ALT |
| rs113443718 | 0.0004 | 4.24 ×10-4 | 0.32 | 0.0003 | 2.53 ×10-4 | 0.41 | 0.0011 | 5.62 ×10-4 | 0.041 |  |
| rs11625650 | 0.0008 | 4.56 ×10-4 | 0.067 | 0.0006 | 2.72 ×10-4 | 0.059 | 0.0022 | 6.02 ×10-4 | 0.00015 | GGT |
| rs11648570 | 0.0024 | 6.30 ×10-4 | 8.50 ×10-5 | 0.0025 | 3.76 ×10-4 | 2.00×10^-^11 | 0.0049 | 8.34 ×10-4 | 1.10 ×10-8 | GGT; ALP; ALT |
| rs11692435 |  |  |  | 0.0008 | 4.36 ×10-4 | 0.038 | 0.0037 | 9.69 ×10-4 | 0.00013 | GGT |
| rs11940694 | 0.0007 | 4.01 ×10-4 | 0.076 | 0.0000 | 2.39 ×10-4 | 0.91 | 0.0054 | 5.31 ×10-4 |  | GGT |
| rs12031875 | 0.0018 | 5.05 ×10-4 | 0.00011 | 0.0003 | 2.97 ×10-4 | 0.3 | 0.0010 | 6.66 ×10-4 | 0.17 | ALT |
| rs1217091 | 0.0013 | 4.96 ×10-4 | 0.0087 | 0.0010 | 2.96 ×10-4 | 0.0017 | 0.0005 | 6.58 ×10-4 | 0.34 |  |
| rs1229984 | 0.0045 | 1.23 ×10-3 | 0.00027 | 0.0092 | 7.32 ×10-4 |  | 0.0122 | 1.62 ×10-3 | 1.90×10^-^14 | GGT; ALP; ALT |
| rs12312693 | 0.0014 | 3.92 ×10-4 | 2.00 ×10-4 | 0.0001 | 2.34 ×10-4 | 0.43 | 0.0023 | 5.18 ×10-4 | 1.10 ×10-5 | GGT; ALT |
| rs12499107 | 0.0014 | 5.72 ×10-4 | 0.017 | 0.0005 | 3.41 ×10-4 | 0.08 | 0.0014 | 7.57 ×10-4 | 0.051 |  |
| rs1260326 | 0.0007 | 3.97 ×10-4 | 0.061 | 0.0061 | 2.36 ×10-4 |  | 0.0140 | 5.24 ×10-4 |  | GGT; ALP |
| rs12646808 | 0.0016 | 4.13 ×10-4 | 0.00018 | 0.0003 | 2.46 ×10-4 | 0.044 | 0.0020 | 5.46 ×10-4 | 0.00015 | GGT;ALT |
| rs12655091 | 0.0001 | 3.90 ×10-4 | 0.94 | 0.0008 | 2.33 ×10-4 | 0.00047 | 0.0004 | 5.15 ×10-4 | 0.42 | ALP |
| rs12795042 | 0.0002 | 4.06 ×10-4 | 0.72 | 0.0002 | 2.41 ×10-4 | 0.47 | 0.0011 | 5.37 ×10-4 | 0.037 |  |
| rs12907323 | 0.0006 | 4.01 ×10-4 | 0.24 | 0.0002 | 2.40 ×10-4 | 0.19 | 0.0008 | 5.31 ×10-4 | 0.16 |  |
| rs13024996 | 0.0001 | 4.02 ×10-4 | 0.96 | 0.0005 | 2.39 ×10-4 | 0.049 | 0.0015 | 5.31 ×10-4 | 0.0082 |  |
| rs13032049 | 0.0003 | 4.33 ×10-4 | 0.36 | 0.0005 | 2.58 ×10-4 | 0.063 | 0.0039 | 5.72 ×10-4 | 1.20×10^-^12 | GGT |
| rs13066454 | 0.0016 | 3.97 ×10-4 | 7.50 ×10-5 | 0.0005 | 2.37 ×10-4 | 0.033 | 0.0017 | 5.25 ×10-4 | 0.0014 | ALT |
| rs13094887 | 0.0010 | 4.24 ×10-4 | 0.013 | 0.0003 | 2.53 ×10-4 | 0.29 | 0.0006 | 5.61 ×10-4 | 0.35 |  |
| rs13107325 | 0.0037 | 7.38 ×10-4 | 2.00 ×10-7 | 0.0006 | 4.40 ×10-4 | 0.093 | 0.0071 | 9.76 ×10-4 | 8.00×10^-^14 | GGT; ALT |
| rs13250583 | 0.0004 | 4.74 ×10-4 | 0.39 | 0.0001 | 2.82 ×10^-^4 | 0.32 | 0.0003 | 6.29 ×10-4 | 0.76 |  |
| rs13383034 | 0.0005 | 4.21 ×10-4 | 0.22 | 0.0006 | 2.51 ×10-4 | 0.016 | 0.0022 | 5.57 ×10-4 | 9.40 ×10-5 | GGT |
| rs13390019 |  |  |  | 0.0006 | 3.43 ×10-4 | 0.077 | 0.0011 | 7.62 ×10-4 | 0.096 |  |
| rs1421085 | 0.0035 | 3.97 ×10-4 | 3.60×10^-^18 | 0.0005 | 2.36 ×10-4 | 0.032 | 0.0018 | 5.25 ×10-4 | 0.0012 | GGT; ALT |
| rs16854020 | 0.0002 | 5.79 ×10-4 | 0.71 | 0.0003 | 3.45 ×10-4 | 0.43 | 0.0014 | 7.65 ×10-4 | 0.065 |  |
| rs1713676 | 0.0005 | 3.89 ×10-4 | 0.22 | 0.0005 | 2.32 ×10-4 | 0.031 | 0.0008 | 5.16 ×10-4 | 0.079 |  |
| rs17177078 | 0.0022 | 8.44 ×10-4 | 0.0051 | 0.0009 | 5.03 ×10-4 | 0.13 | 0.0025 | 1.12 ×10-3 | 0.044 |  |
| rs17665139 | 0.0000 | 5.44 ×10-4 | 0.97 | 0.0001 | 3.24 ×10-4 | 0.83 | 0.0001 | 7.20 ×10-4 | 0.8 |  |
| rs1991556 | 0.0007 | 4.68 ×10-4 | 0.13 | 0.0022 | 2.78 ×10-4 | 2.70×10^-^15 | 0.0026 | 6.20 ×10-4 | 2.50 ×10-5 | GGT; ALP |
| rs2011092 | 0.0008 | 4.09 ×10-4 | 0.051 | 0.0002 | 2.44 ×10-4 | 0.19 | 0.0008 | 5.41 ×10-4 | 0.2 |  |
| rs2071305 | 0.0009 | 4.30 ×10-4 | 0.03 | 0.0015 | 2.56 ×10-4 | 1.40 ×10-8 | 0.0027 | 5.69 ×10-4 | 1.60 ×10-6 | GGT; ALP |
| rs2165670 | 0.0023 | 6.44 ×10-4 | 0.00029 | 0.0006 | 3.84 ×10-4 | 0.22 | 0.0031 | 8.52 ×10-4 | 0.00024 | GGT; ALT; |
| rs2178197 | 0.0018 | 3.93 ×10-4 | 2.60 ×10-6 | 0.0012 | 2.34 ×10-4 | 1.80 ×10-7 | 0.0044 | 5.20 ×10-4 | 2.60E-18 | GGT; ALP; ALT |
| rs227179 | 0.0004 | 3.95 ×10-4 | 0.25 | 0.0007 | 2.32 ×10-4 | 0.0024 | 0.0003 | 5.20 ×10-4 | 0.53 |  |
| rs2277499 | 0.0005 | 4.16 ×10-4 | 0.21 | 0.0004 | 2.48 ×10-4 | 0.11 | 0.0012 | 5.50 ×10-4 | 0.025 |  |
| rs2310752 | 0.0011 | 3.95 ×10-4 | 0.0064 | 0.0006 | 2.32 ×10-4 | 0.009 | 0.0028 | 5.21 ×10-4 | 1.80 ×10-8 | GGT |
| rs2356369 | 0.0010 | 3.89 ×10-4 | 0.0077 | 0.0005 | 2.31 ×10-4 | 0.055 | 0.0001 | 5.15 ×10-4 | 0.72 |  |
| rs2472297 | 0.0006 | 4.42 ×10-4 | 0.23 | 0.0013 | 2.64 ×10-4 | 7.50 ×10-7 | 0.0006 | 5.85 ×10-4 | 0.47 | ALP |
| rs2764771 | 0.0003 | 4.25 ×10-4 | 0.56 | 0.0004 | 2.53 ×10-4 | 0.21 | 0.0004 | 5.63 ×10-4 | 0.36 |  |
| rs281379 | 0.0015 | 3.91 ×10-4 | 0.00014 | 0.0130 | 2.32 ×10-4 | 0 | 0.0114 | 5.17 ×10-4 |  | GGT; ALP; ALT |
| rs2854334 | 0.0005 | 4.02 ×10-4 | 0.23 | 0.0013 | 2.39 ×10-4 | 1.30 ×10-8 | 0.0043 | 5.32 ×10-4 | 4.40×10^-^17 | ALP; GGT; |
| rs28601761 | 0.0082 | 3.98 ×10-4 |  | 0.0082 | 2.37 ×10-4 |  | 0.0090 | 5.27 ×10-4 |  | GGT; ALP; ALT |
| rs28680958 | 0.0005 | 4.70 ×10-4 | 0.29 | 0.0006 | 2.76 ×10-4 | 0.052 | 0.0018 | 6.20 ×10-4 | 0.0023 |  |
| rs34060476 | 0.0055 | 5.72 ×10-4 |  | 0.0038 | 3.41 ×10-4 |  | 0.0161 | 7.56 ×10-4 |  | GGT; ALP; ALT |
| rs35034355 | 0.0006 | 3.89 ×10-4 | 0.083 | 0.0003 | 2.32 ×10-4 | 0.19 | 0.0005 | 5.15 ×10-4 | 0.36 |  |
| rs36052336 | 0.0007 | 8.01 ×10-4 | 0.4 | 0.0015 | 4.78 ×10-4 | 0.0022 | 0.0005 | 1.06 ×10-3 | 0.8 |  |
| rs3748034 | 0.0024 | 5.57 ×10-4 | 6.20 ×10-6 | 0.0001 | 3.32 ×10-4 | 0.83 | 0.0060 | 7.36 ×10-4 | 4.00×10^-^16 | GGT; ALT |
| rs378421 | 0.0016 | 3.98 ×10-4 | 6.20 ×10-5 | 0.0009 | 2.37 ×10-4 | 1.30 ×10-5 | 0.0004 | 5.27 ×10-4 | 0.36 | ALT; ALP |
| rs3803800 | 0.0003 | 4.76 ×10-4 | 0.68 | 0.0009 | 2.83 ×10-4 | 0.00047 | 0.0018 | 6.31 ×10-4 | 0.0047 | ALP |
| rs4092465 | 0.0002 | 4.10 ×10-4 | 0.77 | 0.0021 | 2.45 ×10-4 |  | 0.0002 | 5.43 ×10-4 | 0.97 | ALP |
| rs4548913 | 0.0001 | 4.06 ×10-4 | 0.74 | 0.0006 | 2.42 ×10-4 | 0.011 | 0.0002 | 5.38 ×10-4 | 0.67 |  |
| rs4690727 | 0.0000 | 4.36 ×10-4 | 0.91 | 0.0004 | 2.60 ×10-4 | 0.11 | 0.0006 | 5.76 ×10-4 | 0.32 |  |
| rs4699791 | 0.0002 | 6.57 ×10-4 | 0.78 | 0.0005 | 3.92 ×10-4 | 0.36 | 0.0002 | 8.69 ×10-4 | 0.82 |  |
| rs4794015 | 0.0010 | 3.97 ×10-4 | 0.023 | 0.0006 | 2.36 ×10-4 | 0.0036 | 0.0008 | 5.25 ×10-4 | 0.12 |  |
| rs4815364 | 0.0011 | 4.00 ×10-4 | 0.0034 | 0.0019 | 2.39 ×10-4 | 1.50×10^-^16 | 0.0022 | 5.30 ×10-4 | 7.10 ×10-6 | GGT; ALP |
| rs4842786 | 0.0003 | 3.95 ×10-4 | 0.53 | 0.0000 | 2.35 ×10-4 | 0.59 | 0.0003 | 5.21 ×10-4 | 0.54 |  |
| rs485425 | 0.0011 | 3.90 ×10-4 | 0.0067 | 0.0003 | 2.32 ×10-4 | 0.25 | 0.0004 | 5.16 ×10-4 | 0.29 |  |
| rs4916723 | 0.0000 | 3.98 ×10-4 | 0.88 | 0.0004 | 2.38 ×10-4 | 0.096 | 0.0002 | 5.26 ×10-4 | 0.87 |  |
| rs4938230 | 0.0003 | 5.44 ×10-4 | 0.68 | 0.0001 | 3.24 ×10-4 | 0.99 | 0.0022 | 7.20 ×10-4 | 0.0012 |  |
| rs500321 | 0.0001 | 4.38 ×10-4 | 0.98 | 0.0003 | 2.61 ×10-4 | 0.65 | 0.0004 | 5.80 ×10-4 | 0.39 |  |
| rs55872084 | 0.0007 | 4.54 ×10-4 | 0.15 | 0.0002 | 2.71 ×10-4 | 0.34 | 0.0021 | 6.00 ×10-4 | 0.0011 |  |
| rs55932213 | 0.0001 | 4.54 ×10-4 | 0.96 | 0.0003 | 2.65 ×10-4 | 0.21 | 0.0001 | 6.02 ×10-4 | 0.75 |  |
| rs56337305 | 0.0001 | 4.02 ×10-4 | 0.69 | 0.0003 | 2.39 ×10-4 | 0.13 | 0.0003 | 5.31 ×10-4 | 0.49 |  |
| rs57281063 | 0.0001 | 3.95 ×10-4 | 0.95 | 0.0005 | 2.35 ×10-4 | 0.07 | 0.0010 | 5.21 ×10-4 | 0.04 |  |
| rs58107686 | 0.0005 | 4.12 ×10-4 | 0.21 | 0.0001 | 2.42 ×10-4 | 0.61 | 0.0019 | 5.44 ×10-4 | 0.00082 |  |
| rs60654199 | 0.0021 | 7.76 ×10-4 | 0.0049 | 0.0013 | 4.63 ×10-4 | 0.0032 | 0.0020 | 1.03 ×10-3 | 0.059 |  |
| rs61873510 | 0.0014 | 4.25 ×10-4 | 0.0012 | 0.0008 | 2.53 ×10-4 | 0.00022 | 0.0010 | 5.62 ×10-4 | 0.092 | ALP |
| rs62044525 | 0.0009 | 4.98 ×10-4 | 0.049 | 0.0005 | 2.97 ×10-4 | 0.091 | 0.0020 | 6.59 ×10-4 | 0.0022 |  |
| rs62250685 | 0.0000 | 3.99 ×10-4 | 0.98 | 0.0006 | 2.38 ×10-4 | 0.02 | 0.0002 | 5.29 ×10-4 | 0.94 |  |
| rs6787172 | 0.0001 | 3.91 ×10-4 | 0.73 | 0.0002 | 2.33 ×10-4 | 0.39 | 0.0008 | 5.18 ×10-4 | 0.14 |  |
| rs6951574 | 0.0002 | 3.93 ×10-4 | 0.66 | 0.0003 | 2.34 ×10-4 | 0.13 | 0.0020 | 5.20 ×10-4 | 2.00 ×10-4 | GGT |
| rs7074871 | 0.0002 | 4.43 ×10-4 | 0.61 | 0.0002 | 2.64 ×10-4 | 0.43 | 0.0017 | 5.87 ×10-4 | 0.0039 |  |
| rs7121986 | 0.0005 | 4.00 ×10-4 | 0.23 | 0.0001 | 2.38 ×10-4 | 0.38 | 0.0029 | 5.30 ×10-4 | 1.40 ×10-7 | GGT |
| rs71414193 | 0.0008 | 5.00 ×10-4 | 0.12 | 0.0003 | 2.98 ×10-4 | 0.43 | 0.0007 | 6.61 ×10-4 | 0.17 |  |
| rs7185555 | 0.0011 | 5.34 ×10-4 | 0.041 | 0.0002 | 3.18 ×10-4 | 0.68 | 0.0012 | 7.07 ×10-4 | 0.11 |  |
| rs72859280 | 0.0013 | 1.04 ×10-3 | 0.21 | 0.0004 | 6.17 ×10-4 | 0.64 | 0.0014 | 1.37 ×10-3 | 0.2 |  |
| rs74424378 | 0.0006 | 4.58 ×10-4 | 0.28 | 0.0004 | 2.67 ×10-4 | 0.095 | 0.0004 | 6.06 ×10-4 | 0.66 |  |
| rs748919 | 0.0003 | 4.82 ×10-4 | 0.39 | 0.0006 | 2.87 ×10-4 | 0.011 | 0.0012 | 6.39 ×10-4 | 0.044 |  |
| rs7517344 | 0.0015 | 5.20 ×10-4 | 0.0051 | 0.0005 | 3.05 ×10-4 | 0.12 | 0.0030 | 6.85 ×10-4 | 3.10 ×10-5 | GGT |
| rs7698119 | 0.0000 | 3.97 ×10-4 | 0.89 | 0.0002 | 2.37 ×10-4 | 0.41 | 0.0003 | 5.25 ×10-4 | 0.66 |  |
| rs77165542 | 0.0033 | 1.06 ×10-3 | 0.0025 | 0.0007 | 6.31 ×10-4 | 0.28 | 0.0046 | 1.40 ×10-3 | 0.0029 |  |
| rs780569 | 0.0001 | 4.31 ×10-4 | 0.64 | 0.0002 | 2.53 ×10-4 | 0.5 | 0.0004 | 5.68 ×10-4 | 0.74 |  |
| rs785293 | 0.0001 | 3.96 ×10-4 | 0.83 | 0.0001 | 2.36 ×10-4 | 0.54 | 0.0001 | 5.23 ×10-4 | 0.77 |  |
| rs79139602 | 0.0028 | 1.38 ×10-3 | 0.035 | 0.0003 | 8.26 ×10-4 | 0.59 | 0.0002 | 1.83 ×10-3 | 0.82 |  |
| rs7950166 | 0.0013 | 4.06 ×10-4 | 0.00099 | 0.0004 | 2.41 ×10-4 | 0.072 | 0.0002 | 5.37 ×10-4 | 0.84 |  |
| rs7958704 | 0.0007 | 3.94 ×10-4 | 0.076 | 0.0001 | 2.35 ×10-4 | 0.48 | 0.0019 | 5.21 ×10-4 | 0.00017 | GGT |
| rs823114 | 0.0001 | 3.89 ×10-4 | 0.96 | 0.0015 | 2.29 ×10-4 | 1.70×10^-^10 | 0.0000 | 5.13 ×10-4 | 0.81 | ALP |
| rs828867 |  |  |  | 0.0001 | 2.36 ×10-4 | 0.94 | 0.0006 | 5.24 ×10-4 | 0.3 |  |
| rs9320010 | 0.0002 | 4.02 ×10-4 | 0.81 | 0.0005 | 2.40 ×10-4 | 0.03 | 0.0004 | 5.33 ×10-4 | 0.47 |  |
| rs9607814 | 0.0008 | 4.91 ×10-4 | 0.084 | 0.0003 | 2.94 ×10-4 | 0.27 | 0.0008 | 6.46 ×10-4 | 0.21 |  |
| rs9838144 | 0.0009 | 4.80 ×10-4 | 0.034 | 0.0001 | 2.86 ×10-4 | 0.97 | 0.0001 | 6.36 ×10-4 | 0.84 |  |
| rs988748 | 0.0018 | 4.78 ×10-4 | 0.00021 | 0.0005 | 2.84 ×10-4 | 0.024 | 0.0020 | 6.33 ×10-4 | 0.0026 | ALT |

**Supplementary Table 3- lists the full genotype of each of the C. elegans strains used in the study.**

| Strain | Genotype |
| --- | --- |
| N2 | wild-type |
| FX4735 | *arp-1(tm4735)/mnC1[dpy-10(e128) unc52(e444) nIs190 let-?]) II* |
| VC3166 | *+/mT1 II; arx-3(ok1122)/mT1 [dpy-10(e128)] III* |
| VC4453 | *gop-2(gk5528[loxP + myo-2p::GFP::unc-54 3' UTR + rps-27p::neoR::unc-54 3' UTR + loxP])/+ III* |
| RB954 | *mml-1 (ok849) III* |
| RB796 | *sta-1 (ok587) IV* |
| VC518 | *+/mT1 II; ten-1(ok641)/mT1 [dpy-10(e128)] III* |
| NG2484 | *vab-8 (gm84) V* |
| RB1381 | *abts-1 (ok1566) I* |
| RB809 | *ptl-1 (ok621) III* |
| JD21 | *cca-1(ad1650) X* |
| NL2099 | *rrf-3 (pk1406) II* |

**Supplementary Figure 1**


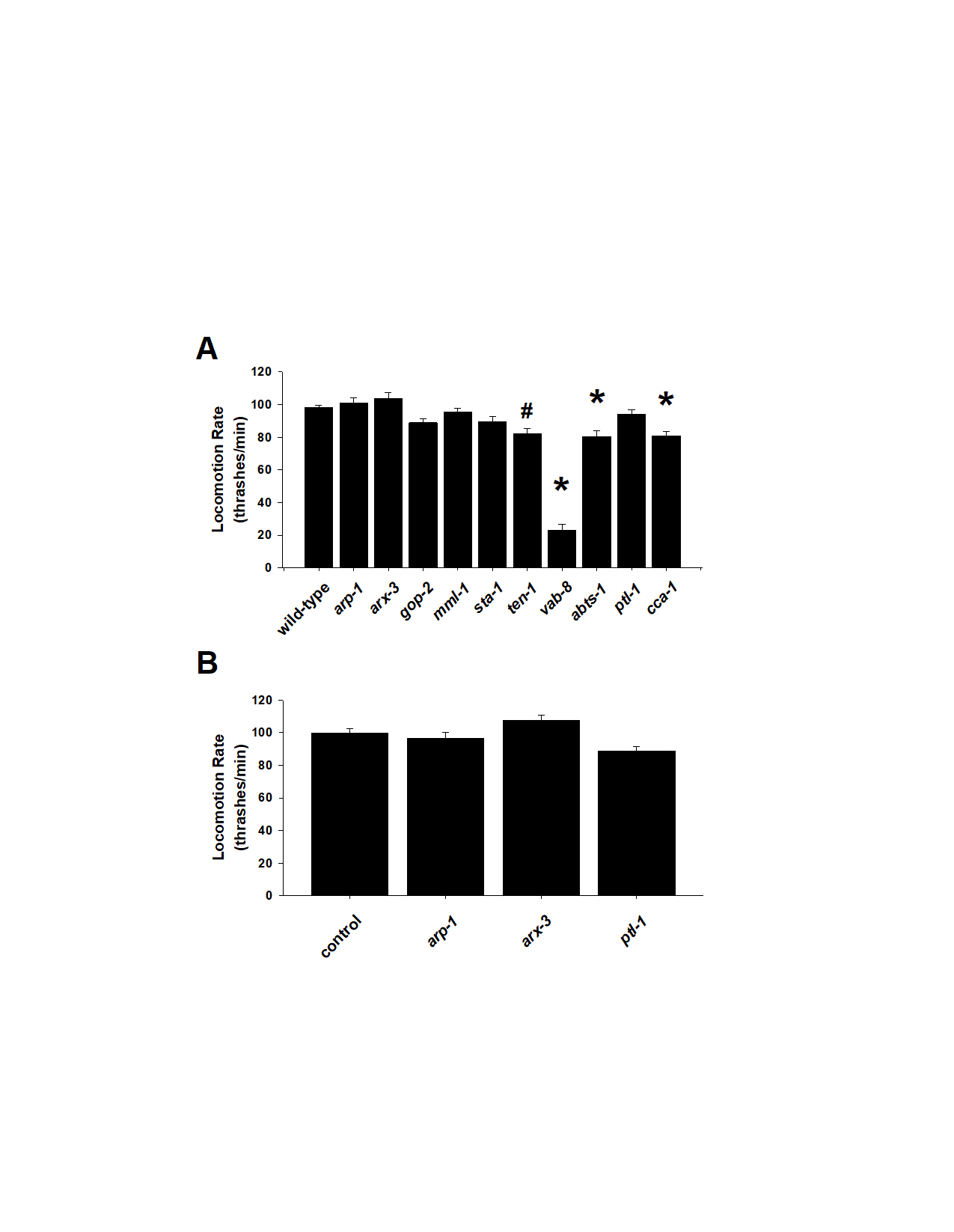


**Supplementary Figure S1- Basal locomotion rates.** (A) Nematodes with *loss-of-function* mutations in worm orthologues of *ACTR1B* (*arp-*1), *ARPC1B* (*arx-3*), *GPN1* (*gop-2*), *MLXIPL* (*mml-1*), *STAT6* (*sta-1*), *TENM2* (*ten-1*), *KIF26A* (*vab-8*), *SLC4A8* (*abts-1*), *MAPT* (*ptl-1*) and *SCN8A* (cca-1) were quantified for locomotion rate (thrashes per minute). In comparison with Bristol N2 wild-type worms, significant differences were identified for *ten-1*, *vab-8*, *abts-1* and *cca-1*. *P<0.01. #P<0.05. (B) Quantification of locomotion rate for worms subjected to RNAi knockdown. In comparison to controls, RNAi knockdown of *arp-1*, *arx-3* or *ptl-1* had no effect on basal locomotion rate.
